# Validation of a Hebrew version of the Early Childhood Oral Health Impact Scale

**DOI:** 10.3389/froh.2025.1543327

**Published:** 2025-05-06

**Authors:** Ofir Gaon, Lena Natapov, Diana Ram, Shlomo Paul Zusman, Avia Fux-Noy

**Affiliations:** ^1^ Faculty of Dental Medicine, Hebrew University of Jerusalem, Jerusalem, Israel; ^2^ Division of Dental Health, Ministry of Health, Jerusalem, Israel; ^3^ Department of Pediatric Dentistry, Hadassah Medical Center, Jerusalem, Israel

**Keywords:** validation, Hebrew, oral health-related quality of life, Early Childhood Oral Health Impact Scale, toddlers

## Abstract

**Background:**

Dental problems such as early childhood caries in toddlers and children can significantly impact their and their family's oral health-related quality of life.

**Aim:**

This study aimed to validate a Hebrew version of the Early Childhood Oral Health Impact Scale (ECOHIS), providing a reliable tool for assessing oral health-related quality of life in toddlers and preschool children.

**Design:**

The ECOHIS questionnaire was translated from English to Hebrew using the “forward–backward translation” method. Two pediatric dentistry specialists evaluated the face and content validity of the Hebrew ECOHIS. Parents of children under 6 years old visiting the pediatric dentistry department at a medical center completed the Hebrew version of the ECOHIS questionnaire and provided their child's personal information. The decayed, missing, and filled teeth (dmft) index was extracted from the dental record.

**Results:**

The study group consisted of 96 children, whose parents participated, including 50 boys and 46 girls, with a mean age of 3.6 years. A positive correlation was found between higher ECOHIS scores and higher dmft indices. No differences were observed between ECOHIS scores and variables such as gender, age, and social subgroups of the participants. The overall scale reliability was high (Cronbach's alpha = .83). Confirmatory factor analysis supported the questionnaire's two-factor structure and indicated a moderate fit to the data.

**Conclusion:**

The Hebrew version of the ECOHIS was found to be valid and reliable for measuring oral health-related quality of life in toddlers and preschool children in Israel.

## 1 Introduction

Oral health-related quality of life (OHRQoL) is a construct that measures an individual's quality of life based on their feelings, subjective perception of oral health, functional abilities, and physical and mental wellbeing, including satisfaction with treatment (1). Dental problems, such as early childhood caries (ECC) in young children, can have a profound impact on both the child's and their family's quality of life (2). ECC is a predictor of future dental caries in later childhood and adulthood (3), and can interfere with eating, speaking, and social development (4). Parents and caregivers may experience emotional distress, including feelings of guilt or responsibility, and financial burdens due to lost workdays and treatment costs (2).

Indices such as the Child-Oral Impact on Daily Performance (cOIDP) and the Oral Health Impact Profile 14 (OHIP-14) have been developed to assess OHRQoL in adults, elderly individuals, and school children. These indices have been validated in Hebrew translations (5, 6). Assessing OHRQoL in young children poses a challenge, as it often relies on subjective feelings that they may find difficult to express verbally (2). In 2007, the Early Childhood Oral Health Impact Scale (ECOHIS) was developed to assess OHRQoL in toddlers and the impact of their oral health on the parent/main caregiver and their families (7). The ECOHIS has been translated into several languages and validated in German, Italian, Spanish, Arabic, and Peruvian-Spanish (8–12).

Despite the implementation of free dental care for all Israeli children under the National Health Insurance law in 2010, ECC remains prevalent, particularly in low socioeconomic communities (13). While only 38.3% of 6-year-old children in Israel are caries-free, a significant 61.7% suffer from dental decay, with an average decayed, missing, and filled teeth (dmft) index of 2.56 (14). Israel's diverse population includes various ethnic and religious groups with distinct sociodemographic and cultural characteristics that can influence the prevalence of dental caries, particularly ECC. For example, some ultra-Orthodox Jewish communities, characterized by larger family sizes, may exhibit lower rates of dental awareness, potentially impacting caries development. Consequently, a significant number of children in these communities require dental treatment under sedation or general anesthesia due to their young age and the complexity of their dental needs (13). This reliance on advanced procedures increases the burden of ECC on children's lives, their families, and the wider community. This study aimed to validate a Hebrew version of the ECOHIS (ECOHIS-He), providing a reliable tool for assessing OHRQoL among preschool children and toddlers in Israel.

## 2 Materials and methods

### 2.1 Study group

The inclusion criteria consisted of parents of healthy children (ASA I) (15) under 6 years old who could read Hebrew and provided informed consent. Parents of children with developmental impairment or chronic disease were excluded due to the children's increased risk for oral diseases, which can significantly impact their health and quality of life. This exclusion was also made to minimize bias related to non-oral health conditions (16). All eligible parents visiting the pediatric dentistry department at a medical center between November 2021 and December 2022 were invited to participate, creating a convenience sample. The department offers dental treatment provided by pediatric dentistry specialists under the National Health Insurance law for children referred from community clinics and private care. The patient population is diverse, representing a range of ethnic, religious, and sociodemographic backgrounds. Participants completed the ECOHIS questionnaire in a self-administered manner and a demographic questionnaire, providing information on their child's age, gender, ID number (for accessing dental records), and social-religious subgroups (Jewish-secular, Jewish-observant, Jewish-ultra-orthodox, or Arab).

The dmft scores were retrieved from the medical records. These scores were recorded by the department's pediatric dentists during the initial dental examination. The examination, conducted using only a dental mirror in accordance with WHO Oral Health Survey Methods (17), was performed with the child seated in a dental chair under artificial light.

### 2.2 Questionnaire translation

The original English version (7) was translated into Hebrew and then back-translated by two independent translators fluent in both languages. Initially, a native Hebrew speaker with expertise in English and experience translating health questionnaires translated the instrument into Hebrew. Then, a native English speaker conducted the back-translation. The back-translation was reviewed by the translators and authors, who confirmed there were no discrepancies in wording. The Hebrew version was pilot-tested with five native-Hebrew-speaking parents, who unanimously reported that the instructions were clear, the vocabulary was accessible, and the response options accurately reflected their experiences. However, they noted a challenge with the questionnaire's structure, as the questions required completing partial sentences. As a result, the questionnaire was restructured to use full sentences. Following the forward–backward translation and revisions based on the pilot group's feedback, the ECOHIS-He was finalized as a simply worded and easy-to-complete questionnaire. Participants from the pilot study were excluded from the final sample.

### 2.3 ECOHIS score calculation

The questionnaire comprises two parts. The first part, the Child Impact section, consists of nine questions assessing the toddler's functioning. The second part, the Family Impact section, consists of four questions regarding the impact of the child's dental health on the family. Parents were asked to indicate the frequency of specific situations occurring from their child's birth to the present, using a Likert scale: never, hardly ever, occasionally, often, very often, or don't know. Numerical values were assigned as follows: never (0), hardly ever (1), occasionally (2), often (3), and very often (4). The “don't know” response was treated as a missing value. Scores were calculated separately for each section. The Child Impact score ranged from 0 to 36, and the Family Impact score ranged from 0 to 16. For the Child Impact section, missing values (up to two) were replaced with the mean score of the remaining responses. For the Family Impact section, a single missing value was replaced with the mean score. Questionnaires with more than two missing values in the Child Impact section or more than one missing value in the Family Impact section were excluded from the study.

### 2.4 Sample size

According to Worthington and Whittaker (18), the adequacy of a sample size for confirmatory factor analysis (CFA) depends on several factors, including the ratio of participants to estimated parameters, with a 5:1 ratio being acceptable and 10:1 being ideal. Therefore, a minimum sample of 65 participants was needed. To ensure adequate power, a larger sample was recruited.

### 2.5 Statistical analysis

Reliability (19): Internal consistency was assessed using the Pearson correlation coefficient to examine the relationship between average scores of conceptually related questions in the two sections (19). Specifically, correlations were calculated between questions 5 and 12 (work/school absences due to dental disease) and questions 7 and 10 (parental and child frustration/stress). In addition, Cronbach's alpha was calculated.

Validity (19): Face and content validity were evaluated by two pediatric dentistry specialists. Construct validity was assessed using convergent and discriminant validity. Convergent validity, the compatibility of logically related variables, was examined by calculating the Pearson correlation coefficient between ECOHIS scores and dmft indices. In addition, *t*-tests were used to compare ECOHIS scores between caries-free toddlers and those with caries experience. Discriminant validity, the incompatibility of logically unrelated variables, was assessed using unpaired *t*-tests to compare ECOHIS scores by gender, Pearson correlation coefficients to examine the relationship between ECOHIS scores and age, and ANOVA to compare ECOHIS scores across different subgroups. Statistical analyses were performed using R in RStudio, with a significance level of *p* < 0.05.

CFA was conducted using AMOS 28 (20) to evaluate the two-factor structure of the ECOHIS-He. Following Hoyle and Panter (21), model fit was assessed using five goodness-of-fit indices: two absolute indices [*χ*^2^ statistic and root mean square error of approximation (RMSEA)] and three incremental indices [normed fit index (NFI), comparative fit index (CFI), and Tucker–Lewis index (TLI)]. RMSEA values below 0.06 in combination with NFI, CFI, and TLI values above 0.95 indicate excellent fit, whereas values below 0.08 and above 0.90, respectively, indicate adequate fit.

### 2.6 Ethics

This study adhered to the principles of the Declaration of Helsinki. Approval was granted by the Committee on Research Involving Human Subjects of the Hebrew University-Hadassah Medical School, Jerusalem, Israel (HMO 0423-21). All participating parents provided informed consent after receiving detailed information about the research methods.

## 3 Results

### 3.1 Study population

Of the 107 parents who visited the pediatric dentistry department with their children during the study period and met the inclusion criteria, 101 (94%) agreed to participate. One participant was excluded due to missing dmft data in their child's medical record. Four questionnaires were excluded because they contained more than two missing responses in the Child Impact section or more than one missing response in the Family Impact section. Consequently, the final study group comprised 96 children.

### 3.2 Sociodemographic characteristics

The mean age of the study population was 3.6 years (SD = 1.31), with a range from 1 to 5.99 years. The sample consisted of 50 boys (52%) and 46 girls (48%). Regarding family subgroup, 50 participants identified as Jewish-ultra-orthodox (52%), 26 as Jewish-observant (27%), 16 as Jewish-secular (17%), and 4 as Arab (4%).

### 3.3 dmft scores

The mean dmft score was 5.86 (SD = 4.78, median = 5.5), with 21 children (22%) caries-free and a maximum dmft score of 20 for one girl.

### 3.4 ECOHIS-He scores

The mean ECOHIS-He score was 12.71 (SD = 7.71), ranging from 0 to 32. The mean section scores were 7.45 (SD = 5.92) for the Child Impact section and 5 (SD = 3) for the Family Impact section. Table 1 presents the mean rating for each question.

**Table 1:** Mean and maximal rating of ECOHIS-He items.

Impact	Item	Mean rating	Maximal rating
Child	1. How often has your child had pain in the teeth, mouth or jaws?	1.53	4
	2. How often has your child had difficulty drinking hot or cold beverages because of dental problems or dental treatments?	0.89	4
	3. How often has your child had difficulty eating some foods because of dental problems or dental treatments?	1.02	5
	4. How often has your child had difficulty pronouncing any words because of dental problems or dental treatments?	0.38	4
	5. How often has your child missed preschool, daycare or school because of dental problems or dental treatments?	0.88	4
	6. How often has your child had trouble sleeping because of dental problems or dental treatments?	1.02	4
	7. How often has your child been irritable or frustrated because of dental problems or dental treatments?	1.13	4
	8. How often has your child avoided smiling or laughing because of dental problems or dental treatments?	0.33	4
	9. How often has your child avoided talking because of dental problems or dental treatments?	0.23	5
Family	10. How often have you or another family member been upset because of your child's dental problems or dental treatments?	1.56	4
	11. How often have you or another family member felt guilty because of your child's dental problems or dental treatments?	1.33	4
	12. How often have you or another family member taken time off from work because of your child's dental problems or dental treatments?	1.48	4
	13. How often has your child had dental problems or dental treatments that had a financial impact on your family?	0.76	4

### 3.5 Reliability assessment

Ratings for questions 7 (mean = 1.13, SD = 1.21) and 10 (mean = 1.56, SD = 1.22) were strongly correlated [*r*(94) = 0.41, *p* < 0.0001]. Similarly, ratings for questions 5 (mean = 0.88, SD = 0.88) and 12 (mean = 1.48, SD = 1.49) were strongly correlated [*r*(94) = 0.43, *p* < 0.0001].

The Child Impact subscale demonstrated good internal consistency, with a Cronbach's alpha of 0.84. However, the Family Impact subscale showed low reliability (α = 0.53). The overall scale reliability was good (α = 0.83).

### 3.6 Validity assessment

Content validity and face validity: Two pediatric dentistry specialists reviewed the Hebrew version and confirmed that the index is a suitable tool for measuring OHRQoL. They affirmed that it accurately reflects all elements characterizing toddlers’ OHRQoL related to their oral health, including symptoms, function, social relationships, psychological impact, and family impact.

Convergent validity: ECOHIS-He scores were higher in children with caries experience. The mean score for caries-free children (*n* = 21) was 9.66 (SD = 6.09), while the mean score for children with caries experience (*n* = 75) was 13.57 (SD = 7.93). A *t*-test revealed a statistically significant difference between the two groups: t(41) = 2.4194, *p* = 0.020. A statistically significant weak positive correlation was observed between ECOHIS-He scores and dmft scores [*r*(94) = 0.226, *p* = 0.027]. When analyzing the correlation between dmft and the Child and Family Impact sections separately, a non-significant positive linear relationship was observed for Child Impact scores (*r* = 0.190, *p* = 0.062), while a statistically significant positive linear relationship was found for Family Impact scores (*r* = 0.208, *p* = 0.046).

Discriminant validity: An unpaired t-test showed no statistically significant difference [t(90) = 0.29, *p* = 0.771] between the ECOHIS-He scores of boys (*n* = 50, mean = 12.94, SD = 7.26) and girls (*n* = 46, mean = 12.47, SD = 8.25). A Pearson correlation coefficient test revealed no statistically significant correlation (*r* = 0.125, *p* = 0.227) between ECOHIS-He scores and participant age. An ANOVA test indicated no significant difference [F(2, 89) = 0.75, *p* = 0.47] between mean ECOHIS-He scores across subgroups: Jewish-secular (*n* = 16, mean = 10.18, SD = 7.36), Jewish-observant (*n* = 26, mean = 12.26, SD = 6.7), and Jewish-ultra-orthodox (*n* = 50, mean = 12.8, SD = 7.75). The Arab group (*n* = 4, mean = 24.75, SD = 4.9) was excluded from the analysis due to its small sample size.

**Figure 1:**
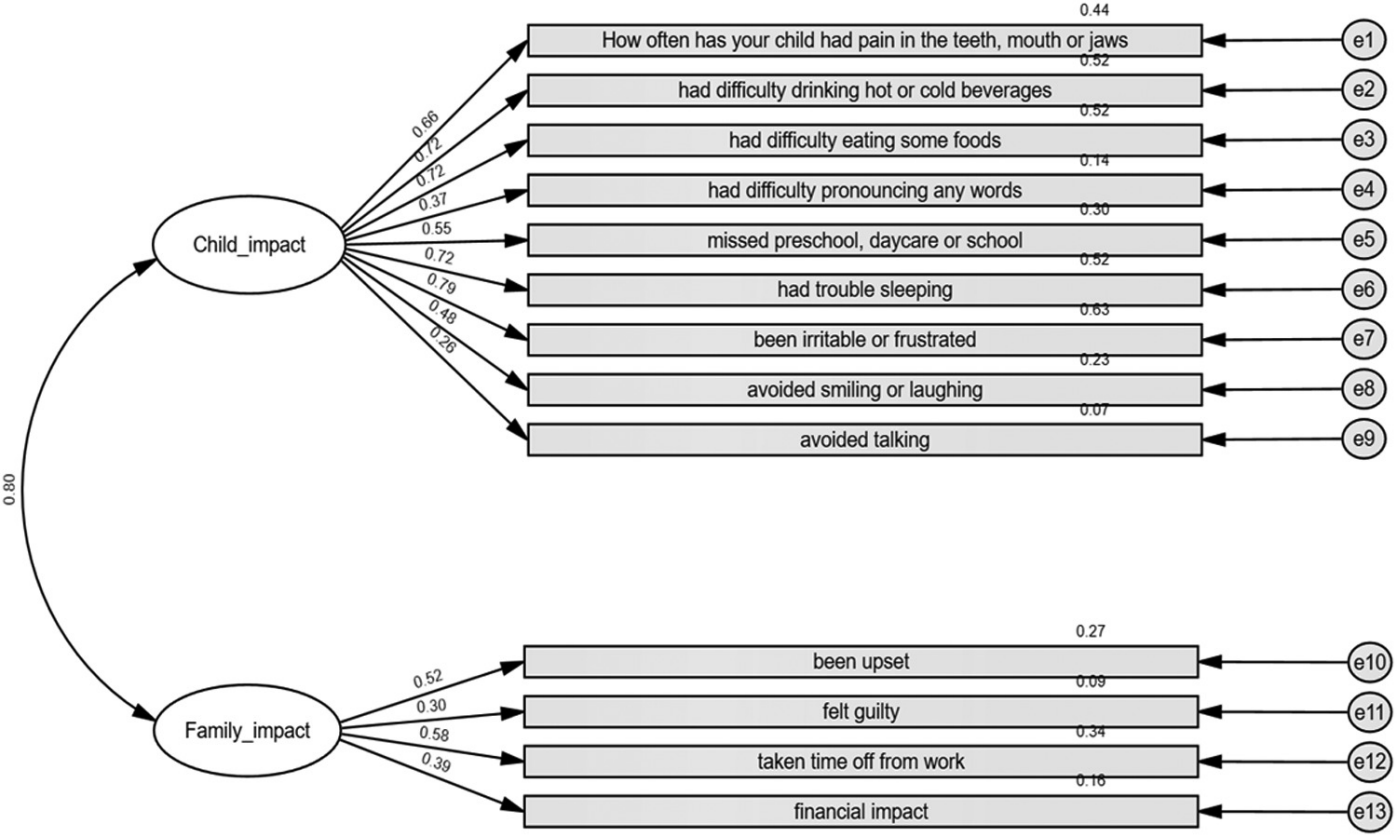
Confirmatory factor analysis of the ECOHIS-He items. The values in the figure are standardized coefficients.

The CFA results partially supported the two-factor structure of the questionnaire, indicating a moderate fit to the data. The significant chi-square statistic [*χ*^2^(64) = 147.05, *p* < 0.001] suggested a lack of perfect model fit. The incremental fit indices also indicated marginal model fit: RMSEA = 0.12, NFI = 0.67, CFI = 0.78, and TLI = 0.73. Figure 1 displays the standardized factor loadings for the model. The Child Impact factor (nine items) showed strong standardized loadings for most items, whereas the Family Impact factor (four items) exhibited weaker loadings overall (ranging from 0.30 to 0.58). A strong and significant correlation was found between the two factors (*r* = 0.80, *p* < 0.001).

## 4 Discussion

Extensive evidence supports the validity and reliability of various OHRQoL measures in assessing the burden of ECC on children's lives, their families, and communities. Due to linguistic and cultural differences, OHRQoL measures must be adapted for use in diverse languages and cultures to facilitate cross-national and cross-cultural research on the global impact of ECC (22). This study found that the Hebrew version of the ECOHIS is a reliable and valid tool for measuring OHRQoL in toddlers among Hebrew-speaking parents.

The mean ECOHIS-He score for our study population was 12.71. This value is comparable to values found in studies conducted in Slovenia (23), Saudi Arabia (12), and Lithuania (24), with 15.3, 14.6, and 13.4, respectively, suggesting a similar level of OHRQoL impact in these populations. Conversely, a significantly lower mean ECOHIS score of 4.15 was reported in Thailand (25). This difference can likely be attributed to the Thai population's notably lower mean dmft of 1.63, indicating better overall dental health and thus less impact on their quality of life. Studies in Mexico (26), France (27), and Germany (8) also reported lower mean ECOHIS scores, ranging from 3.2 to 6.1. However, the absence of reported dmft values in these studies makes it difficult to directly assess the potential influence of caries experience on these scores. In contrast, higher mean ECOHIS scores were observed in Peru with 17.02 (11) and Iran with 25.7 (28). Again, without corresponding dmft data, it is challenging to determine the specific factors contributing to these elevated scores. While dmft appears to be a significant factor, it is also possible that cultural, socioeconomic, and access-to-care differences between these populations may contribute to the observed variations in ECOHIS scores. Further investigations are needed to explore these potential influences.

Although the dmft index reflects a child's caries experience, it does not indicate the presence of active decay at the time of examination. Active decay can negatively impact quality of life through pain, eating difficulties, and sleep disturbances. Thus, focusing solely on the “d” (decayed) component might yield different results. However, it is crucial to note that the ECOHIS questionnaire assesses the impact of both dental problems and treatment, meaning that treated caries also affects ECOHIS scores. The “f” (filled) and “m” (missing) components can also have implications. For example, esthetic concerns may arise from anterior teeth extractions (29), from the appearance of posterior stainless-steel crowns, or from the black stain of silver diamine fluoride (30).

In the Child Impact section of the current study, the highest mean scores were observed for items related to dental pain (teeth, mouth, or jaw), difficulty eating some foods, sleep disturbances, and irritability/frustration. Similarly, in the Family Impact section, the highest mean scores were associated with family members’ distress (feeling upset and guilty) and work absenteeism. Notably, dental pain consistently emerged as a prominent concern, mirroring findings in other studies (11, 26, 31–34). Furthermore, difficulties with eating and child irritability/frustration were also frequently reported in previous research (11, 31, 32, 34). Parental upset and guilt were similarly recognized as having significant impacts on families across diverse studies (11, 31–34). It is noteworthy that these specific items consistently showed high mean scores across various cultural contexts, suggesting a universal aspect to the impact of oral health on children and their families.

No difference was found in ECOHIS scores among the ultra-orthodox, observant, and secular Jewish participants. As all participants were recruited from the pediatric dentistry department at a single medical center, it is likely that they reside within the same geographic area in the Central District of the country. Therefore, it is reasonable to assume that factors relevant to oral health, such as fluoride levels in drinking water and access to dental services, are similar across all subgroups.

Although more parents from the Arab subgroup were invited to participate, they could not meet the inclusion criterion of Hebrew literacy. To accurately assess the Arab population in Israel, an Arabic version of the ECOHIS should be utilized. An Arabic version was validated in Saudi Arabia in 2017 (12). However, if the dialect in Israel significantly differs, validating the ECOHIS in the local Arabic dialect of the Israeli Arab community is recommended.

The study had a few limitations. First, the sample was relatively small and drawn from a single medical center, which may limit the generalizability of the findings. The sample size of 96 participants was selected to align with established recommendations for CFA (18). For CFA models of moderate complexity, such as this study with two latent factors and 13 observed variables, a sample size of approximately 100 participants is considered appropriate (18). Although the sample size in this study slightly falls below this threshold, it meets the recommended participant-to-item ratio of at least 5:1, supporting the stability and interpretability of the model. The sample size was comparable to other ECOHIS validation studies in languages such as Italian (9), Chinese (35), and Peruvian Spanish (11). In addition, participants were recruited from a dental clinic, irrespective of their visit's purpose (e.g., dental trauma, emergency, or scheduled appointment). This recruitment strategy could potentially introduce bias in ECOHIS-He responses (36, 37). However, the significant difference in ECOHIS scores between caries-free children and children with caries experience and the correlation observed between ECOHIS-He scores and dmft indices support the measure's validity. Future studies should include population-based studies with larger samples, and investigate the ECOHIS's responsiveness to changes in clinical conditions and patient-reported outcomes. All of these studies are important for validating the instrument in the Israeli context. In conclusion, the Hebrew version of the ECOHIS was found to be valid and reliable. Additional research is required to validate these findings and confirm the tool as an appropriate measure of oral health-related quality of life of children aged 0–6 years in the Hebrew-speaking population of Israel.

## Data Availability

The raw data supporting the conclusions of this article will be made available by the authors, without undue reservation.
